# Invasive Mucormycosis in a Patient With Liver Cirrhosis: Case Report and Review of the Literature

**DOI:** 10.5812/hepatmon.10858

**Published:** 2013-08-11

**Authors:** Hussien Elsiesy, Mohamed Saad, Mahmoud Shorman, Samir Amr, Faisal Abaalkhail, Almoutaz Hashim, Waleed Al Hamoudi, Mohamed Al Sebayel, Khalid Selim

**Affiliations:** 1 Department of Liver Transplantation, King Faisal Specialist Hospital and RC, Riyadh, Saudi Arabia; 2 Department of Medicine, National Liver Institute, Menoufia, Egypt; 3 Department of Medicine, King Fahd Specialist Hospital-Dammam, Dammam, Saudi Arabia; 4 Department of Pathology, King Fahd Specialist Hospital-Dammam, Dammam, Saudi Arabia; 5 Nazih Zuhdi Transplant Institute, INTEGRIS Baptist Medical Center, Oklahoma, USA

**Keywords:** Amputation, Gangrene, Liver Cirrhosis, Mucormycosis

## Abstract

**Introduction:**

Cutaneous Mucormycosis is a rare opportunistic infection caused by Zygomycetes class of fungi that is often fatal, requiring aggressive local control as well as systemic therapy. Few cases of mucormycosis were described in patients with liver cirrhosis, mostly rhino-orbital. To our knowledge, only two cases of upper extremity involvement was reported in cirrhosis while a few cases were reported in the post-transplant setting. We report herein the third case of upper extremity mucor infection in the setting of liver cirrhosis.

**Case Presentation:**

We described a rare case of forearm infection originating in a traumatic intravenous access portal in a 25 year-old woman with liver cirrhosis secondary to autoimmune hepatitis.

**Discussion:**

She developed acute on chronic liver failure during the last trimester of pregnancy, which was terminated. Painful, erythematous lesion was noted on her right forearm in the area of intravenous access, which later became necrotic. Extensive debridement was done and histopathological examination confirmed the diagnosis of mucormycosis. The patient started on Amphotericin B. Her condition continued to deteriorate and ended up with above elbow amputation followed by right shoulder disarticulation. She died two days later due to multi-organ failure. In conclusion, forearm mucromycosis in liver cirrhosis can be fatal.

## 1. Introduction

Mucormycosis is a rare opportunistic fungal infection characterized by infarction and necrosis of host tissues that is resulted from invasion of the vasculature by hyphae (1) The most common clinical presentation of mucormycosis is rhino-orbital-cerebral infection. It can also cause pulmonary, gastrointestinal, cutaneous, renal, and disseminated diseases (2). Diabetes is the most common risk factor, founded in 36% of all cases, followed by hematologic malignancies (17%), and solid organ or hematopoietic stem cell transplantation (12%) (3). A systematic literature review of Medline was performed (01/1970-12/2012) including the search terms: Liver cirrhosis, Mucormycosis, amputation, gangrene. Additional manual review of the reference lists of the identified case reports. The medical literature on this subject revealed a small number of cases (n = 17 cases including this case) reported in twelve papers (4-15), most with fatal outcome.

## 2. Case Presentation

A 25-year-old woman with liver cirrhosis secondary to autoimmune hepatitis diagnosed at a local hospital presented with fatigue, painless jaundice and lower limb swelling for two weeks, there was no evidence of hypertension or protenuria excluding the possibility of pre-eclampsia or eclampsia. There was no history of ascites, spontaneous bacterial peritonitis or hepatic encephathy. Pregnancy was terminated at 27th week at the local hospital. She was on 40 mg of Prednisolone daily for possible autoimmune hepatitis, soon after, she developed steroid induced diabetes. She was referred to our hospital after 6 weeks of the intial presentation with two episodes of hematemesis, tachycardia (heart rate 120) and blood pressure of 84/50. The patient was admitted to ICU and started on octreotide and Piperacillin/Tazobactam. Gastroscopy revealed gastric varices and bleeding was endoscopically controlled. During admission, liver function tests worsened, and transjugular liver biopsy showed established liver cirrhosis with no obvious active pathology. We started tapering the steroids slowly after being on 40 mg for 4 weeks with no improvement of jaundice without evidence of active inflammation on liver biopsy. During admission, she developed painful, erythematous lesion on her right forearm at the IV access area that became necrotic and spread quickly and increased in size over a period of 24 hours. The lesion included the skin and subcutaneous tissue and was flecked with tiny black spots. Vital signs: temp 37.4ºC, BP 128/80 mmHg, pulse 66 /min, RR 20 /min, SaO2 100% on room air.

Chest was clear to auscultation and cardiovascular exam was normal. Abdomen was soft and lax, distended with ascites but no tenderness. Extremities showed mild lower limb edema. Her investigations showed a white cell count 14.24 x 103/mm3 with Neutrophils of 86%, hemoglobin 9.2 g/dl, and platelets of 103000 /mm3 , LFT: total bilirubin 457 umol/L, direct 321, alkaline phosphatase 430U/L, ALT 174U/L, AST 248U/L, gamma GT 227, total protein 51g/L, albumin 18g/L, PT 19.4 and INR was 1.7, PTT 58, renal function test was within normal, fasting glucose 9.8 mmol/L. ESR was 2, Blood culture showed no growth after 5 days.

MRI of the right upper extremity showed inflammatory changes through the anterior compartment of the forearm suggestive of fasciitis.

She was taken to the operating room for debridement after 24 hours, delay was due to anesthesia issues in this high risk patients. At this point after 18 days of admission, Prednisolone dose reached 20 mg daily and antibiotic changed from Piperacillin/Tazobactam to Imipenem when the culture results from tissue biopsy grew Klebsiella Pneumonia extended spectrum b lactamase producer (ESBL).

**Figure 1.**
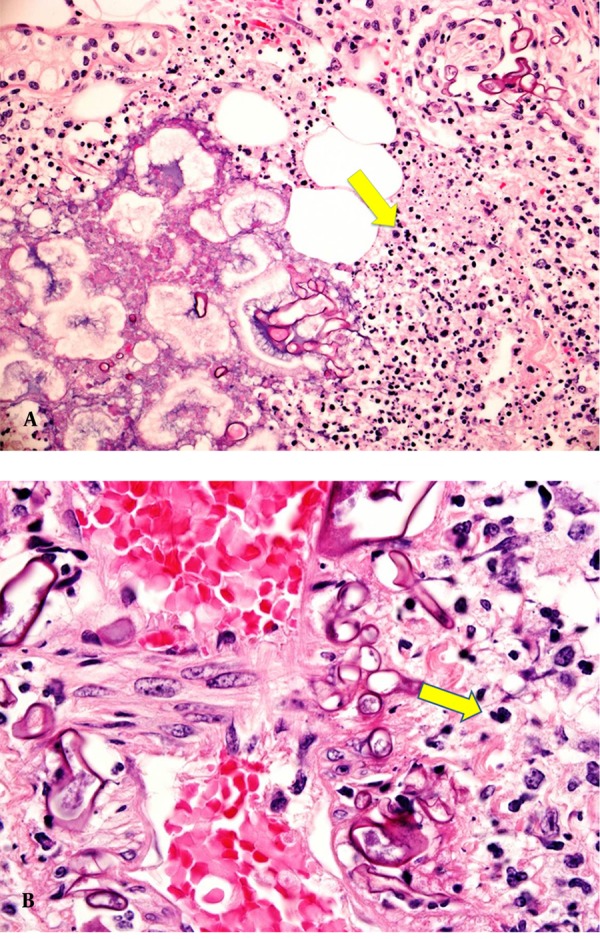
A) Extensive fat necrosis from soft tissue of forearm with neutrophilic infiltrate and several irregular broad fungal hyphae. (PAS stain x200) B) Irregular thick fungal hyphae with rare septa invading the walls of blood vessels, a feature of mucor fungal infection. (PAS stain x400)

Histopathological examination of the debrided soft tissue revealed necrotic subcutaneous fat and skeletal muscle fibers invaded by broad hyphae, irregularly branched, with rare septations suggestive of mucormycosis (Figure 1A and 1B). There were no clinical symptoms or signs of visceral dissemination of infection to warrant further imaging or biopsy. The patient started on Amphotericin B (Abelcet®) 300mg IV OD after 20 days of admission and was continued on imipenem. In spite of surgical debridement, her condition continued to deteriorate and ended up after 6 days with above elbow amputation, tissue-cultures for both bacteria and fungi were negative. Amphotericin B was stopped after amputation as it was considered that the source of infection was eliminated but was restarted again 3 days after discontinuation with imipenem continued all through with the addition of vancomycin. The patient continued to deteriorate and the stump showed signs of disseminated infection, which proved to be invasive mucormycosis infection. Right shoulder disarticulation done but she passed away 2 days later due to multi-organ failure after 35 days in the hospital.

## 3. Discussion

There are more than 900 cases of mucormycosis reported in the literature (3), Diabetes is the most common risk factor but it has been described with hematological malignancy, solid tumors, bone marrow and solid organ transplantation and recipients of deferoxamine therapy (3). Sixteen cases of mucormycosis are reported in patients with liver cirrhosis (4-15). The majority of these cases are rhino-orbital. Three cases including this case were upper extremity and one case was gastric involvement (Table 1). 

**Table 1.** All the Cases of Mucormycosis Patients With Liver Cirrhosis and Their Outcomes

Patients no	Age/ Gender	Child Pugh class	Etiology	Cirrhosis	Comorbidity Complication	Involved Area	Treatment	Outcome	Reference	Year
**1**	44/Female	B	NR a	NR	LC^a^	Ethmoid sinus, orbit	None	Died	(9)	1993
**2**	53/Male	B	HCV^a^	HCC^a^	LC, DM^a^	Not done	None	Died	(10)	1998
**3**	58/F	C	NR	NR	LC, DM, RI^a^	Paranasal sinuses, orbit, cerebral	Liposomal AmB^a^	Died	(7)	2003
**4**	39/M	B	HCV	None	LC, DM	Maxillary, ethmoid & sphenoid sinus,cerebral	Surgery/AmB	Died	(4)	2007
**5**	57/M	C	HCV	PSE^a^, SBP^a^, HCC	LC	Not done	AmB	Died	(4)	2007
**6**	55/M	C	HBV	PSE, SBP, HRS HCC^a^	LC	Maxillary, and ethmoid sinus	None	Died	(4)	2007
**7**	15/F	C	AIH	PSE, SBP	LC, DM, steroid therapy	Maxillary sinus	None	Died	(4)	2007
**8**	53/M	C	HCV	None	LC, DM	Maxillary, ethmoid and sphenoid sinuses, orbit, cerebral	Surgery (Orbit enucleation & endoscopic debridement of sinuses)/ AmB	Died	(4)	2007
**9**	35/M	C	HCV	SBP, HRS	LC, DM	Maxillary ethmoid and sphenoid sinus, orbit	AmB	Died	(4)	2007
**10**	63/F	B	HCV	None	LC	Maxillary, ethmoid sphenoid sinuses and orbit	Surgery/AmB, Liposomal AmB	Alive	(12)	2009
**11**	42/M	C	HBV	PSE	None	Sphenoid, ethmoid, and maxillary sinuses	AmB	Died	(5)	2010
**12**	59/F	C	Alcohol	PSE	LC, HTN	Paranasal sinuses, orbits, bilateral infratemporal fossa, right cavernous sinuses, bilateral optic nerve		Died	(6)	2011
**13**	65/M	B	HCV	None	DM, HTN	Maxillary and ethmoid sinuses	Surgery, AmB, liposomal AmB, itraconazole, posaconazole	Alive	(11)	2012
**14**	47/M	C	HCV	Gastric mucormycosis, Cirrhosis	HTN, DM	Gastric Ulcer	Amphotericin B	Died	(14)	2012
**15**	38/F	C	Alcohol	PSE, SBP, HRS	LC, steroids	Right Forearm	Amputation	Died	(13)	2007
**16**	48/F	NR	Alcohol	PSE	NR	Right Forearm	Local excision	Died	(15)	2010
**17**	25/F	C	AIH	Variceal bleeding	LC, steroids	Right Forearm	Amputation, AmB	Died	This case	2013

^a^Abbreviations: AmB, amphotericin B; DM, diabetes mellitus; HBV, Hepatitis B virus; HCV, Hepatitis C virus; HCC, hepatocellular carcinoma; HTN, hypertension; PSE, portosystemic encephalopathy; SBP, spontaneous bacterial peritonitis; HRS, hepatorenal syndrome; LC, liver cirrhosis; RI, renal insufficiency; NR, Not reported

To our knowledge, there are only two cases of upper limb mucormycosis which are reported previously in association with cirrhosis (13, 15). Moreover, cases of limb involvement in the post-transplant setting are rare (16). Raizman et al., reported in 2007 the first case of upper extremity mucormycosis in the setting of liver cirrhosis in a 38 year old lady with alcoholic cirrhosis, who was on steroids. She developed gangrene and underwent amputation. The diagnosis was confirmed after the patient death (13). Wollstein et al, reported another case in 2010 of a 48 year old lady with decompensated alcoholic cirrhosis and hepatic encephalopathy admitted with pneumonia and septic shock, developed of right forearm mucormycosis treated with local excision, and eventually she died (15). In our case, the diagnosis was made relatively early and the patient was started on antifungal therapy few days after the initial presentation. As in the case reported in 2007, our patient died despite above elbow amputation followed by shoulder disarticulation together with antifungal therapy. There was an initial insult to the forearm with an IV access in our case. In some cases a traumatic incident preceded the infection. In a case described by Raizman et al., the infection seemed to have started in an arterial line and caused necrosis of the hand distally (13). Another case of localized mucormycosis was described following an intramuscular injection of corticosteroids (17). Another case was reported after use of contaminated dressing (18).

Mucormycosis in liver cirrhosis has been reported, including the present case, in 17 patients: 9 males and 8 females. Cirrhosis was due to HCV in 8 patients; HBV in 2; autoimmune hepatitis (AIH) in 2; and alcoholic liver disease (ALD) in 3. The etiology was not reported in 2 cases. Mucormycosis was not reported in Child A, there were 11 cases with Child C and 5 with Child B and in one case, the Child’s score was not reported (15). The two survivals of mucormycosis in patients with liver cirrhosis were in patients with Child’ B cirrhosis (11, 12). Eight cases have DM as co-morbidity while 3 patients were on steroids.

The mortality is high despite aggressive surgical debridement and proper antifungal and antibacterial coverage, there was a relative delay in diagnosing the fungal infection but treatment started once the cultures came back positive. The outcome may be determined mainly by underlying risk factor; mucormycosis in diabetics has 60% to 90% the survival, where it is 20%-50% in Leukemia. In Cirrhosis it is 11.7% (2 out of 17 patients).

In conclusion; mucormycosis carries high mortality in patients with liver cirrhosis despite aggressive treatment, the three cases with limb involvement, the gastric mucromycosis and 11 out of 13 cases of rhino-orbital involvement died. From this case and in reviewing the literature, it seems that underlying risk factors of the patient determine the final outcome more than the particular treatment provided.
